# Formate, acetate, and propionate as substrates for sulfate reduction in sub-arctic sediments of Southwest Greenland

**DOI:** 10.3389/fmicb.2015.00846

**Published:** 2015-08-24

**Authors:** Clemens Glombitza, Marion Jaussi, Hans Røy, Marit-Solveig Seidenkrantz, Bente A. Lomstein, Bo B. Jørgensen

**Affiliations:** ^1^Department of Bioscience, Center for Geomicrobiology, Aarhus UniversityAarhus, Denmark; ^2^Department of Bioscience, Arctic Research Center, Aarhus UniversityAarhus, Denmark; ^3^Department of Geoscience, Centre for Past Climate Studies, Aarhus UniversityAarhus, Denmark; ^4^Section for Microbiology, Department of Bioscience, Aarhus UniversityAarhus, Denmark

**Keywords:** volatile fatty acids, porewater, thermodynamics, Gibbs energy, minimum energy requirement, acetate diffusion, energy turnover, turnover rates

## Abstract

Volatile fatty acids (VFAs) are key intermediates in the anaerobic mineralization of organic matter in marine sediments. We studied the role of VFAs in the carbon and energy turnover in the sulfate reduction zone of sediments from the sub-arctic Godthåbsfjord (SW Greenland) and the adjacent continental shelf in the NE Labrador Sea. VFA porewater concentrations were measured by a new two-dimensional ion chromatography-mass spectrometry method that enabled the direct analysis of VFAs without sample pretreatment. VFA concentrations were low and surprisingly constant (4–6 μmol L^−1^ for formate and acetate, and 0.5 μmol L^−1^ for propionate) throughout the sulfate reduction zone. Hence, VFAs are turned over while maintaining a stable concentration that is suggested to be under a strong microbial control. Estimated mean diffusion times of acetate between neighboring cells were <1 s, whereas VFA turnover times increased from several hours at the sediment surface to several years at the bottom of the sulfate reduction zone. Thus, diffusion was not limiting the VFA turnover. Despite constant VFA concentrations, the Gibbs energies (ΔG_r_) of VFA-dependent sulfate reduction decreased downcore, from −28 to −16 kJ (mol formate)^−1^, −68 to −31 kJ (mol acetate)^−1^, and −124 to −65 kJ (mol propionate)^−1^. Thus, ΔG_r_ is apparently not determining the *in-situ* VFA concentrations directly. However, at the bottom of the sulfate zone of the shelf station, acetoclastic sulfate reduction might operate at its energetic limit at ~ −30 kJ (mol acetate)^−1^. It is not clear what controls VFA concentrations in the porewater but cell physiological constraints such as energetic costs of VFA activation or uptake could be important. We suggest that such constraints control the substrate turnover and result in a minimum ΔG_r_ that depends on cell physiology and is different for individual substrates.

## Introduction

Mineralization of buried organic matter drives microbial activity and element cycles in sub-seafloor sediments (Reeburgh, 1983; Arndt et al., 2013). The amount and quality of this sedimentary organic matter determines the rates of microbial processes and the microbial abundance (Kallmeyer et al., 2012; Røy et al., 2012; Algora et al., 2013; Glombitza et al., 2013; Orcutt et al., 2013). The initial step in the degradation of high molecular weight organic matter in anoxic sediments is the hydrolytic breakdown of polymers by exoenzymes excreted by heterotrophic bacteria. This produces smaller compounds (mono- and dimers) which can be taken up by fermentative bacteria (e.g., Capone and Kiene, 1988). Fermentation does not result in a net oxidation of the organic matter but produces volatile fatty acids (VFAs), small alcohols, amines, CO_2_, H_2_ etc. The VFAs are quantitatively important intermediates and are turned over in the terminal steps of organic matter mineralization (e.g., sulfate reduction) by which they serve as electron donors (Middelburg, 1989).

VFA concentrations in sediment porewater thus reflect a balance between VFA generation and consumption, both of which take place in the same sediment zone. Many studies have analyzed the terminal mineralization steps by measuring the turnover of the electron acceptor, in particular of sulfate (Jørgensen, 1982; Burdige, 1993; Orcutt et al., 2013). However, the intermediates are equally important because they link the organic matter decomposition to the terminal oxidation processes. Under steady state conditions the concentrations of the intermediates are low and reflect a close coupling of fermentation and terminal oxidation. However, when environmental conditions change the transition can cause an imbalance with elevated concentrations. For example Hoehler et al. (1998) reported that acetate (and H_2_) concentrations increased after depletion of sulfate in incubation experiments with coastal marine sediments. The loss of the dominant electron acceptor, sulfate, resulted in an increase in fermentation products because they were initially degraded at a lower rate. After a significant time lag the steady state concentrations of the intermediates were restored by microorganisms under methanogenic conditions.

The role of specific VFAs as electron donors in organic matter mineralization has been studied by radiotracer and inhibitor incubation experiments (Christensen and Blackburn, 1982; Ingvorsen et al., 1984; Shaw and McIntosh, 1990; Finke et al., 2006). The earlier analytical methods for VFAs in marine porewater required sample pretreatment such as derivatisation or distillation and they often lacked sufficient sensitivity to quantify the low *in-situ* concentrations. We recently developed a very sensitive analytical approach based on two-dimensional ion chromatography-mass spectrometry (2D IC-MS) (Glombitza et al., 2014). This approach enables the direct quantification of several C_1_–C_5_ VFAs, lactate and pyruvate from marine porewater without sample pretreatment and with a detection limit of 0.1–0.5 μmol L^−1^. We used this 2D IC-MS method to obtain high resolution depth profiles of VFAs in the porewater from 6-m deep sediment cores retrieved from the sub-arctic Godthåbsfjord system in Southwest Greenland as well as outside the fjord on the Greenland shelf in the northeastern Labrador Sea. A pycnocline separating the saline bottom water from the glacial fresh water at the surface prevents the water from mixing and maintains a constant bottom temperature of 2°C (Mortensen et al., 2011). This stable environment is ideal to investigate how VFA concentrations are balanced at steady state.

The aim of our study was thus to understand the controls on *in-situ* porewater concentrations of VFAs in relation to the turnover of carbon and energy in the sulfate reduction zone of marine sediments. To relate the *in-situ* VFA concentrations to the carbon and energy turnover in the sediment, porewater concentrations of VFAs, sulfate, sulfide, and DIC were used to calculate the Gibbs energy of sulfate reduction utilizing individual VFAs as electron donors. We calculated the turnover rates of individual VFAs from experimentally measured sulfate reduction rates. We also evaluated the influence of VFA diffusion in the sediment on the microbial availability of VFAs and discuss the potential for thermodynamic control of *in-situ* porewater concentrations. A more detailed analysis of microbial activity and diversity in relation to sediment age and properties will be presented in a subsequent study (Jaussi et al., unpublished).

## Materials and methods

### Study area and sample material

#### Study area

**Figure 1:**
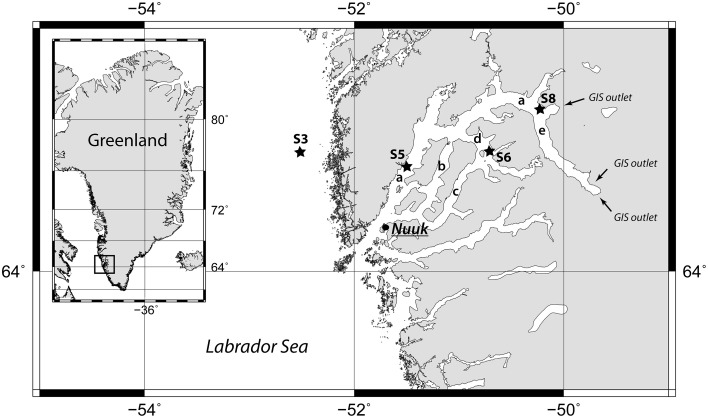
**Map of the Godthåbsfjord region including the four coring stations marked with black stars**. The arrows indicate the three outlets of the Greenland Ice Sheet (GIS) to the Kangersuneq. The letters indicate, a, main fjord (Nûp Kangerdlua); b, Qôrnup Suvdlua; c, Ũmánap Suvdlua; d, Kapisigdlit Kanderdluat; e, Kangersuneq. Map created with GMT5 (http://gmt.soest.hawaii.edu/).

The Godthåbsfjord (Figure 1) is a complex, sub-arctic fjord system in southwest Greenland consisting of a main fjord named Godthåbsfjord or Nûp Kangerdlua and several connected side branches, Qôrnup Suvdlua and Umánap Suvdlua, Kapisigdlit Kanderdluat, and the Kangersuneq which receives three glacier outlets from the Greenland Ice Sheet (GIS). The capital of Greenland, Nuuk (64°10′N, 51°44′W), is located at the mouth of the fjord. The combined area of the Godthåbsfjord is 2013 km^2^ with a volume of 525 km^3^. Mean water depth is 260 m with several sills (Mortensen et al., 2011). The fjord system is characterized by the inflow of saline, well-oxygenated water from the Labrador Sea (West Greenland Current water) as a subsurface/bottom current filling the deep basins of the fjord, and the outflow of less saline water, primarily derived from glacier meltwater, which exits the fjord as a surface current (Buch, 2002).

In August 2013, 6-m long gravity cores were retrieved at four stations outside and inside of the fjord system during a cruise with *R/V Sanna*. Additionally, two shorter cores were retrieved by a Rumohr Corer (Meischner and Rumohr, 1974) at Station 3 and 6 (Table 1). *Station 3* (Figures 1, S3), is located outside of the fjord on the continental shelf in the Labrador Sea. At this site, there is a strong northward flow of the West Greenland Current. *Station 5* (Figures 1, S5) is located in the main fjord (Figure 1, a). A number of turbidites were identified in this core. *Station 6* (Figures 1, S6) is located in the Kapisigdlit Kanderdluat fjord (Figure 1, d) and *Station 8* (Figures 1, S8) is located in close proximity of the northernmost outlet of the GIS in the Kangersuneq (Figure 1, e). This site is today characterized by turbid water and significant sediment influx from the melting glacier. CTD profiles were measured at all sites and confirmed the presence of oxygenated bottom water at marine salinity (*S* = 35). *In-situ* temperatures measured at the bottom of the cores and were similar to the bottom water temperatures (Station 3: 4°C, Stations 5, 6, and 8: 2°C).

**Table 1:** **List of stations and cores with coring position, water depth, and core length**.

**Station**	**Core name**	**Latitude (N)**	**Longitude (W)**	**Water depth (m)**	**Core length (cm)**	**Device**
Station 3	SA13-ST3-17R-B	64°26.743′	52°47.3664′	498.2	55	RC
Station 3	SA13-ST3-20G	64°26.7425′	52°47.6486′	498.2	587	GC
Station 5	SA13-ST5-30G	64°25.3479′	51°30.6209′	622.4	607	GC
Station 6	SA13-ST6-35R	64°29.1406′	50°42.4669′	411.6	83	RC
Station 6	SA13-ST6-40G	64°29.0604′	50°42.3240′	389	562	GC
Station 8	SA13-ST8-47G	64°40.7078′	50°17.4672′	475.8	569	GC

*RC, Rumohr Corer; GC, Gravity Corer*.

#### Porewater sampling

Samples for porewater analyses were taken from all cores by Rhizon soil-moisture samplers (Seeberg-Elverfeldt et al., 2005) that had been previously cleaned by filtering through 50 mL Milli-Q water and stored in sealed gas-tight bags to minimize contamination by VFAs from air and equipment. This treatment was previously shown in blank samples to reduce contamination by VFAs from the porous membrane of the Rhizon samplers to below detection levels (Glombitza et al., 2014). Porewater samples were obtained by pushing the soil-moisture samplers through 3-mm wide holes drilled in the core liner. The first 2 mL were used for chloride, sulfate, sulfide, and dissolved inorganic carbon (DIC) analysis. The following 1 mL was sampled for VFA analysis in baked (450°C, 5 h) glass vials and stored frozen at −80°C for 3 months before analysis. Porewater samples were taken every 5–10 cm in the Rumohr cores and every 25 cm in the gravity cores.

#### Sampling for cell counting

Sediment for cell counts was retrieved with sterile 2.5 mL syringes with cut-off tips through windows cut in the core liners. One cm^3^ sediment was transferred into centrifuge tubes with 4 mL filter-sterilized NaCl solution (30 g L^−1^) amended with paraformaldehyde (2% w:v). The sample was shaken to form a homogenous slurry. Samples were stored at 4°C until analysis in the laboratory.

#### Samples for sulfate reduction rate determination

Small sub-cores for sulfate reduction rate determination were taken from sediment cores in sterile 5 mL plastic syringes with a cut-off tip through cut windows immediately after porewater sampling. The windows were cut a few cm away from the porewater sampling positions at the same depths as the porewater samples but carefully avoiding cross contamination. The samples were kept in air-tight bags with AnaeroGen™ O_2_ scrubbers (OXOID, Thermo Scientific; do not leak H_2_) at the *in-situ* bottom water temperature (2°C) until incubation with ^35^S-labeled sulfate tracer, immediately after subsampling the core (typically within 2–4 h).

### Analytical methods

#### VFA concentrations in porewater

VFA concentrations were measured by two-dimensional ion chromatography-mass spectrometry using a Dionex ICS3000 ion chromatograph coupled to an MSQ Plus (Thermo Scientific) mass spectrometer. The method is described in detail in Glombitza et al. (2014). Briefly, by this method the first IC dimension is used to separate the inorganic ions, such as chloride, from the VFAs. The VFAs are trapped on a concentrator column and subsequently separated in the second IC dimension. The column for the first dimension was a Dionex IonPac™ AS24 and for the second dimension was a Dionex IonPac™ AS11HC (both Thermo Fisher Scientific). Prior to IC-MS analysis the samples were defrosted and filtered through disposable Acrodisc® 13 mm IC syringe filters (pore size 0.2 μm) that had been flushed with 10 mL Milli-Q water directly before use. The first 0.5 mL of porewater after filtration was discarded and the next 0.5 mL was used for IC-MS analysis. All samples were measured undiluted and without further treatment.

Detection limits for the individual VFAs are all between 0.1 and 0.5 μmol L^−1^. For a detailed discussion of analytical and statistical parameters (detection limits, sensitivity, accuracy, and precision) of all analytes see Glombitza et al. (2014). Because the samples were measured without dilution or pre-treatment other than filtration, potential contamination may only derive from the sampling device (Rhizon), the syringe filter or the sampling vial. This was all evaluated carefully in Glombitza et al. (2014) and detailed instructions for sampling and sample preparation (such as baking the vials and effective cleaning and storage of filters and Rhizon samplers) were presented to minimize the contamination below the analytical detection limit. The background (peak areas deriving from the instrumental analytical procedure itself, i.e., peaks deriving from formate and acetate background in eluent accumulating in the trap column) was determined by repeated blank measurement runs without sample injection every 5–10 measurements and used to correct the peak areas in the sample measurements.

#### Inorganic ion concentrations in porewater

*Sulfate and chloride* concentrations were measured by suppressed ion chromatography with an ICS2500 system (Dionex) equipped with an eluent generator (EG50) and KOH eluent generator cartridge (EGC III KOH). The column was a Dionex IonPac™ AS18 operated at 30°C. KOH concentration started at 20 mmol L^−1^ and was raised to 32 mmol L^−1^ at the end of the analysis run at 15 min. *Hydrogen sulfide* (sum of H_2_S, HS^−^ and S^2−^) was determined spectrophotometrically at 670 nm (FLUOstar Omega, BMG Labtech GmbH, Orthenberg, Germany) on zinc-preserved porewater samples by the methylene blue method (Cline, 1969; Reese et al., 2011). *DIC* was measured immediately after the cruise on headspace-free porewater samples stored at 4°C. Samples were transferred to sealed exetainers and acidified with 85% (v:v) phosphoric acid. After 24 h of equilibration time, the produced CO_2_ was measured from the headspace of the exetainer by a Delta V™ isotope ratio mass spectrometer (IRMS, Thermo Scientific).

#### Cell abundance

Microbial cells were quantified according to the protocol of Kallmeyer et al. (2008) with a few modifications. Briefly, the slurries were first treated with a filter-sterilized solution of NaCl (30 g L^−1^), detergent mix and methanol and then mixed for 60 min on a Thermomixer comfort® (Eppendorf AG, Hamburg) at 1200 rpm and 18°C. After a first density centrifugation with filter-sterilized Nycodenz solution (50% w/v) to separate cells from sediment particles, the slurries were further treated mechanically by sonication. The sonication probe (SONOPULS HD 2070, Bandelin Berlin, Germany) was placed directly in the samples and pulsed 3 times for 10 s at the minimum adjustable level (10%) including a 30 s break between the individual pulses. After a second density centrifugation with Nycodenz solution, the pooled cell extracts were filtered through 25 mm black polycarbonate filters (GTBP, 0.2 μm-pore size) and washed with 2 mL of TE buffer (pH 8.0). After drying, the filters were stained with DAPI-CV-mounting solution for 20 min [DAPI stain (1:100), 4:1 CitifluorTM (Citifluor Ldt, England)-Vectashield® mounting medium (Vector laboratories)]. At least 400 cells were counted over a minimum of 12 fields of view in an epifluorescence microscope (Axiovert 200M Zeiss, Germany). An additional acid treatment was included for slurries containing carbonates, before the chemical treatment (Kallmeyer et al., 2008). Those slurries were amended with 500 μL carbonate dissolution mix [aqueous solution with high acidity and moderate pH (4.6) containing 0.43 mol L^−1^ glacial acetic acid and 0.43 mol L^−1^ sodium acetate] for minimum 1 h (until CO_2_ bubbles were no longer observed), followed by three consecutive washing steps with NaCl (30 g L^−1^).

#### Sulfate reduction rate determination

Sulfate reduction rates (SRRs) were determined by incubation with ^35^S-labeled sulfate in undisturbed sediments samples (Jørgensen, 1978; Røy et al., 2014). Carrier-free (without non-radioactive sulfate) ^35^${\text{SO}}_{4}^{2-}$ (10 μL, 10 kBq μL^−1^) was injected with a Hamilton gas tight syringe in the center of each sub-core. The samples were incubated for 12 h at 2–3°C in the dark in sealed air-tight bags with AnaeroGen™ O_2_ scrubber (OXOID, Thermo Scientific). To terminate the incubation, the samples were frozen in the bags at −20°C. In order to calculate sulfate reduction rates, the total reduced inorganic sulfur (TRIS) was separated from sulfate by a cold chromium distillation described in detail in Kallmeyer et al. (2004) including modifications and recommendations by Røy et al. (2014). Before the distillation, the samples were thawed and suspended in 5 mL 20% (w:v) Zinc-Acetate (ZnAc) solution. Na_2_S (200 μL, 0.5 mol L^−1^) was added to the reaction flask as a sulfide carrier. At the end of the distillation, the distillate in the ZnAc trap was transferred into 15 mL scintillation liquid (Ecoscint XR, National diagnostics, Atlanta, GA, USA). The radioactivity of the total sulfur (a_TOT_) and in the reduced sulfur fraction (a_TRIS_) was measured in a liquid scintillator counter (Packard Tri-Carb 2900 TR liquid scintillation analyzer). Samples were counted for 30 min. Blank samples, which were transferred to ZnAc (20% w:v) before tracer injection, were used to determine the background. Sulfate reduction rates (SRR) were calculated according to Kallmeyer et al. (2004) (Equation 1):

(1) $$\begin{array}{l} SRR=\left[{SO}_{4}^{2-}\right]\times \phi \times \frac{a_{TRIS}}{a_{TOT}}\times \frac{1}{t}\times 1.06 \end{array}$$

where $\left[{SO}_{4}^{2-}\right]$ is the porewater sulfate concentration, ϕ is the porosity, *a*_*TRIS*_ the radioactivity of the reduced sulfur fraction, *a*_*TOT*_ the total sample radioactivity, *t* the incubation time and 1.06 the correction factor for the estimated microbial isotopic fractionation of sulfur during sulfate reduction (Jørgensen and Fenchel, 1974). Porosity was calculated from the weight loss of 2 cm^3^ of wet sediment during drying (100°C until constant weight). Mean values were calculated when duplicate SRRs were determined at the same depth.

### Calculation of the Gibbs energy

The Gibbs energy of a reaction (ΔG_r_) is constrained by the chemical and physical environment in which the reaction takes place, namely the pressure, the temperature, and the activities of the educts and products. The temperature was 2°C while the pressure ranged from 4.0 to 6.3 MPa according to the water depth (Table 1). We used a mean pressure of 5 MPa for calculations. The ΔG_r_ is calculated from the standard Gibbs energy of reaction, ${\Delta \text{G}}_{\text{r}}^{0}$, according to Equation (2):

(2) $$\begin{array}{l} △{\text{G}}_{\text{r}}={△\text{G}}_{\text{r}}^{0}+\text{R}\;\text{ln}Q_{r} \end{array}$$

where *R* (0.008314 kJ mol^−1^ K^−1^) is the universal gas constant, *T* (in K) is the temperature, and *Q*_*r*_ is the reaction quotient of the specific reaction. The standard Gibbs energies of reaction, ${\Delta G}_{r}^{0}$, are a function of temperature and pressure. Values of ${\Delta \text{G}}_{\text{r}}^{0}$ (*T, p*) are calculated using the revised-HKF (Helgeson-Kirkham-Flowers model) equations of state (Helgeson et al., 1981; Tanger and Helgeson, 1988; Shock et al., 1992) and the software package SUPCRT92/OBIGT (Johnson et al., 1992). Thermodynamic properties were taken from Shock and Helgeson (1988), Shock (1995), and Shock et al. (1997). The standard state thermodynamic properties of the species are listed in the Supplementary Table 1. The reaction quotient *Q*_*r*_ is calculated as the product (Π) of the activities of the species in the specific reaction (Equation 3):

(3) $$\begin{array}{l} Q_{r}=\underset{\text{i}}{\prod }{a_{\text{i}}}^{\nu_{\text{i}}}=\left(\frac{\underset{\text{i}}{\prod }{a_{\left(\text{product}\right)}}^{\nu_{\text{i}}}}{\underset{\text{i}}{\prod }{a_{\left(\text{educt}\right)}}^{\nu_{\text{i}}}}\right) \end{array}$$

where *a* denotes the activities of the reaction participants (educts and products), and ν the stoichiometric coefficient of the *i*-th educt or product. The activities were approximated by multiplying the measured concentrations of the species by the activity coefficients. Activity coefficients were calculated using an extended version of the Debye-Hückel equation (Helgeson, 1969) and the Geochemists Workbench® (www.gwb.com) software for an ionic strength of *I* = 0.7 and a temperature of 2°C (275 K). Activity coefficients used for the calculations are given in the Supplementary Table 1. The Gibbs energy of reaction calculated by Equation 2 is used to calculate the energy per mol of a specific substrate (Δ*G*_*r, i*_) by dividing Δ*G*_*r*_ by the stoichiometric coefficient ν_*i*_ of the respective *i*-th substrate (Equation 4):

(4) $$\begin{array}{l} △G_{r,i}=\frac{{△G}_{r}}{\nu_{i}} \end{array}$$

## Results

### Formate, acetate, and propionate

**Figure 2:**
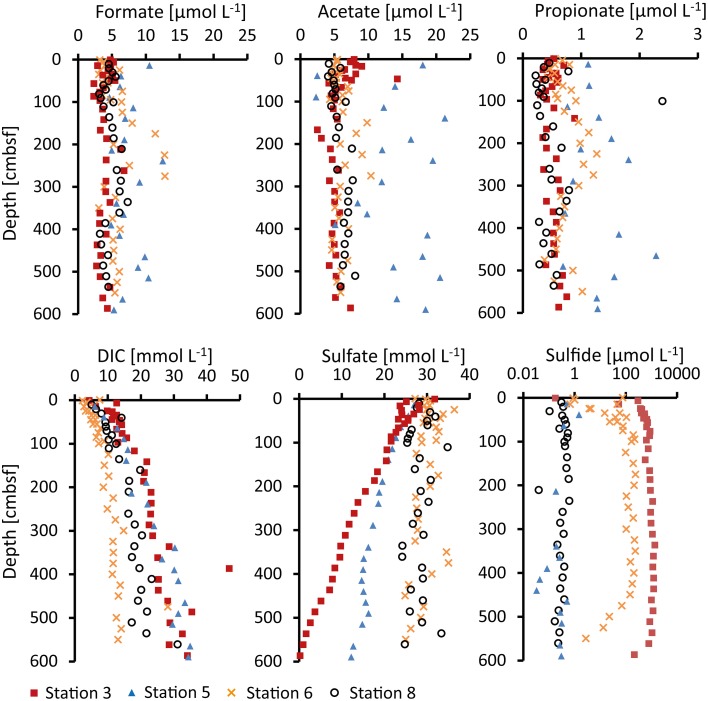
**Concentrations of formate, acetate, propionate, dissolved inorganic carbon (DIC), sulfate and sulfide (H_2_S + HS^−^) measured in the porewater**.

Formate, acetate, and propionate were detected in the porewater of all four stations. Butyrate and valerate were constantly below our detection limit (0.13 and 0.18 μmol L^−1^). Depth profiles of the measured VFA concentrations are shown in Figure 2.

Formate concentrations were nearly constant with depth at Station 3 and 8 with average values of 4–5 μmol L^−1^. At Station 6, the concentrations were mostly similar (average of 5 μmol L^−1^), with the exception of 100–300 cmbsf where they were slightly higher and more scattered (average of 9 μmol L^−1^). Concentrations in the porewater of Station 5 were slightly higher and more scattered throughout the whole core with concentrations between 5 and 13 μmol L^−1^ (average 7 μmol L^−1^).

Acetate concentrations at Station 3 decreased slightly from 9 to 5 μmol L^−1^ through the upper 100 cm below which the concentrations remained at 5–6 μmol L^−1^. At Stations 6 and 8 average concentrations were 6 μmol L^−1^ with exception of the 100–300 cmbsf interval at Station 6 where acetate concentrations were around 8 μmol L^−1^ and more scattered. As for formate, the acetate concentrations in the porewater of Station 5 were generally slightly higher and more scattered (2.5–21 μmol L^−1^, average 14 μmol L^−1^).

Propionate concentrations were about an order of magnitude lower than formate and acetate in all cores but also rather constant with depth. At Stations 3 and 8, average concentrations were 0.5 μmol L^−1^. At Station 6, they were slightly higher (0.7 μmol L^−1^), in particular in the 100–300 cmbsf depth interval with average concentrations of 1 μmol L^−1^. Like for formate and acetate, propionate concentrations at Station 5 were generally higher and more scattered with values of 0.7–1.8 μmol L^−1^ (average 1.3 μmol L^−1^).

### Inorganic ions

Measured concentration profiles of inorganic ions in the porewater of all cores are shown in Figure 2. *DIC* increased with depth up to 35 mmol L^−1^ at the bottom of the core at Stations 3 and 5. At Station 8, the increase in DIC with depth reached ~20 mmol L^−1^. At Station 6, the concentrations of DIC remained constant at depths beneath 100 cmbsf (11–13 mmol L^−1^). *Sulfate* was present to the bottom of the cores at all four stations. Sulfate concentrations at Station 3 decreased from surface values of 28–0.2 mmol L^−1^ at the bottom of the core. At Station 5, the sulfate concentration profile was less steep and reached 12–15 mmol L^−1^ at the bottom of the core. The concentrations at Stations 6 and 8 were nearly constant throughout the cores with values of around 28 mmol L^−1^. *Sulfide* was highest at Station 3 where the concentration increased from <1 μmol L^−1^ at the surface to about 1 mmol L^−1^ at 200 cmbsf below which it remained constant. At Station 6, sulfide increased in the upper 100 cm to 100–200 μmol L^−1^ and was constant from 100–450 cmbsf below which it decreased to 1 μmol L^−1^. Stations 5 and 8 showed low sulfide throughout the cores (<1 μmol L^−1^, Figure 2, note the logarithmic scale for sulfide concentrations). *Chloride* concentrations in the porewater remained constant with depth (460–520 mmol L^−1^).

### Sulfate reduction rates

**Figure 3:**
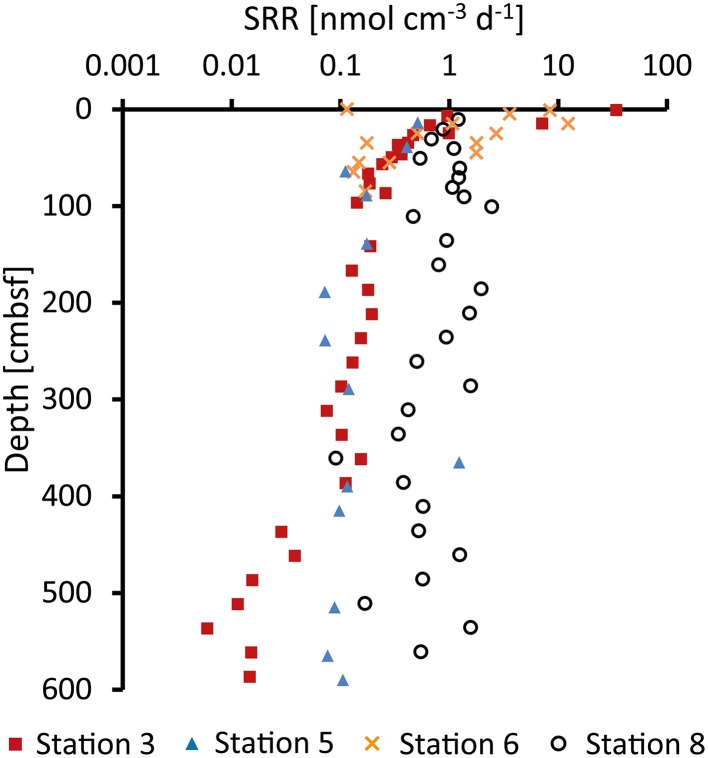
**Sulfate reduction rates (SRR) measured by incubation experiments with ^35^S-labeled sulfate**.

At Station 3, sulfate reduction rates were highest, 35 nmol cm^−3^ d^−1^, near the sediment surface (Figure 3). At this station, the rates showed a >1000-fold decrease downcore and reached 0.01 nmol cm^−3^ d^−1^ at the bottom of the core. SRRs at Station 5 were relatively constant with depth (~0.1 nmol cm^−3^ d^−1^). At Station 6, SRRs were high near the top and decreased to 0.1 nmol cm^−3^ d^−1^ at 100 cmbsf. Below this depth SRRs were below detection. At Station 8, in contrast, SRRs were generally high (around 1 nmol cm^−3^ d^−1^) throughout the sediment core.

### Cell abundance

**Figure 4:**
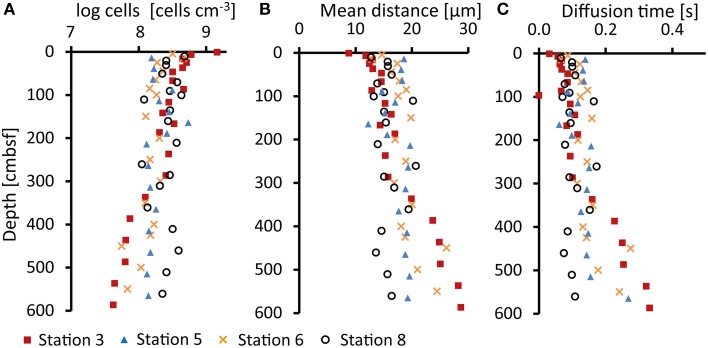
**(A)** Cell abundance (log cells cm^−3^) determined in sediments samples after cell extraction, staining (DAPI), and counting under a fluorescence microscope, **(B)** Calculated mean distances (μm) between nearest cells, and **(C)** mean acetate diffusion times (seconds) between nearest cells calculated from cell abundances.

Cell numbers at Stations 3 and 6 were typical for costal sediments (Parkes et al., 2000; Kallmeyer et al., 2012) with up to nearly 10^9^ cells cm^−3^ in the uppermost samples (<10 cmbsf) and a decrease down core (Figure 4A). At Stations 5 and 8, cell numbers remained rather constant throughout the cores at 10^8^ cells cm^−3^.

## Discussion

Our study focused on potential controls on *in-situ* porewater concentrations of formate, acetate and propionate which are key intermediates in subsurface microbial metabolism. We measured VFA concentrations in the porewater of sediment cores from the sub-arctic Godthåbsfjord and the adjacent continental shelf. In order to understand what determines the *in-situ* concentrations of these metabolic intermediates we measured sulfate reduction rates by radiotracer incubations to estimate the VFA turnover rates. We determined the abundance of cells in the sediments to evaluate the influence of VFA diffusion times between the cells. Additionally, we analyzed the concentrations of sulfate, sulfide, and DIC in the porewater to calculate Gibbs energy for sulfate reduction in order to identify energetic limitations for this metabolic process.

### Porewater VFA concentrations

Porewater VFA concentrations in the sulfate reduction zone were in the low micromolar (formate and acetate) to sub-micromolar (propionate) range. In coastal marine sediments, higher acetate concentrations (several 10 s to 100 μmol L^−1^) have been reported earlier, by using a vacuum distillation technique to separate the VFA from porewater chloride and by subsequent gas chromatographic analysis (e.g., Christensen and Blackburn, 1982). Other studies that applied ion chromatographic analysis after vacuum distillation (Parkes and Taylor, 1983) or derivatisation and subsequent HPLC analysis (Albert and Martens, 1997) reported similar porewater acetate concentrations in the sulfate reduction zone as reported here (Ansbaek and Blackburn, 1980; Balba and Nedwell, 1982; Jørgensen and Parkes, 2010; Vandieken and Thamdrup, 2013).

It is striking that the porewater VFA concentrations show very little variability with depth and that the profiles are very similar between the stations. The slightly higher and more fluctuating VFA concentrations at Station 5 were most likely caused by fluctuating sedimentation, indicated by numerous turbidities throughout the core. The amount and quality (reactivity) of sedimentary organic matter usually control the rates of mineralization in the sediments (Jørgensen, 1982; Røy et al., 2012; Algora et al., 2013; Glombitza et al., 2013). From the observed decrease in measured sulfate reduction rates (Figure 3) it can be concluded that the organic matter mineralization rates are highest at the top 10's of cm of sediment and decrease with depth. Accordingly, we assume a decrease of VFA production rates by fermentation.

In general, the *in-situ* porewater concentrations measured in all cores were constant and presumably in steady state. As the mineralization rates, and thus the production rates of the individual VFAs, decrease strongly with depth, the VFA concentrations are apparently under strict control by the consumers. There appears to be a lower threshold concentration below which the microbial uptake of VFAs is inhibited. The threshold concentrations are similar for formate (4.0–4.5 μmol L^−1^) and acetate (5.4–5.9 μmol L^−1^), with only slightly lower concentration for formate, whereas the threshold concentration for propionate (0.5 μmol L^−1^) is an order of magnitude lower. As propionate may be further fermented to acetate and H_2_, the low concentration of propionate could be due to control by fermentation instead of the terminal oxidation by sulfate reduction. If the observed VFA concentrations are indeed threshold levels controlled by the uptake by sulfate reducers or other heterotrophic bacteria in the sulfate zone, then concentrations might be different in other sediment zones (e.g., in the underlying zone of methanogenesis). Although the relatively constant VFA concentrations found in the sulfate reduction zone suggest uptake control it is not clear what determines the specific levels of the observed VFA concentrations. Are the apparent threshold concentrations determined by kinetic or energetic properties of the cellular uptake and metabolism, by the chemical properties of VFAs in the porewater, or by other mechanisms?

### VFA turnover rates

Turnover rates of VFAs by sulfate reduction can be estimated from measured sulfate reduction rates based on the stoichiometry of their reaction with sulfate (formate:sulfate = 4:1, acetate:sulfate = 1:1, propionate:sulfate = 0.57:1, see Equations 6–9, Section Gibbs Energy of Sulfate Reduction) and an estimation of the contribution of the individual VFAs to feed the sulfate reducers. There are only few studies that have estimated the contribution of different VFAs as electron donors for sulfate reduction in marine sediments. To the best of our knowledge, no study has yet estimate the *in-situ* contribution of formate to sulfate reduction. Sørensen et al. (1981) measured the initial accumulation of acetate, propionate, butyrate, and hydrogen in coastal anoxic sediment during incubation with molybdate (${\text{MoO}}_{4}^{2-}$) added to inhibit sulfate reduction. Accumulation rates of the VFAs and hydrogen were expected to represent the turnover rates of these substrates by sulfate reduction in the un-inhibited sediment. The authors attributed 10% of the sulfate reduction to hydrogen, 40–50% to acetate, 10–20% to propionate and 10–20% to butyrate. In a similar experiment, Christensen (1984) found a mean contribution of 65% acetate, 14% propionate, 8% butyrate, and 6% isobutyrate.

The turnover of acetate in sediment was also determined by incubation experiments with ^14^C-labeled acetate added and subsequent measurement of the concentrations of acetate and the decrease of the labeled substrate (Christensen and Blackburn, 1982; Shaw et al., 1984; Shaw and McIntosh, 1990). These experiments showed mostly higher rates than those determined by the molybdate-amendment method and sometimes rates even exceeded the measured sulfate reduction rates or the total mineralization rates estimated from DIC or ${\text{NH}}_{4}^{+}$ production (Ansbaek and Blackburn, 1980; Christensen and Blackburn, 1982). It was suggested that a part of the measured acetate was complexed or adsorbed and thus represented an acetate pool which was less available to microorganisms than the free ^14^C-labeled acetate (Parkes et al., 1984). Thus, earlier estimates based on the ^14^C-incubation method seem to overestimate *in-situ* turnover rates.

Finke et al. (2006) estimated the contribution of individual substrates to sulfate reduction in Arctic fjord sediments using two different methods: (1) inhibition of sulfate reduction by the addition of selenate (${\text{SeO}}_{4}^{2-}$) and measuring the initial increase of substrate concentrations, and (2) incubations with ^14^C-labeled substrates that were previously equilibrated in sterilized porewater to achieve potential complexion of the labeled acetate and to mimic *in-situ* conditions. The turnover rates determined by both methods were similar. A contribution of 40% acetate, 8% propionate, 1.3% butyrate, and 3% lactate to the total sulfate reduction was estimated.

In order to estimate acetate and propionate turnover rates, we have chosen values for the respective contributions to sulfate reduction of 40% acetate and 8% propionate as published in Finke et al. (2006). We consider these data to be realistic estimates based on the discussion above. Furthermore, the relative differences between the published data are small in relation to the main conclusions drawn in the following discussion.

**Figure 5:**
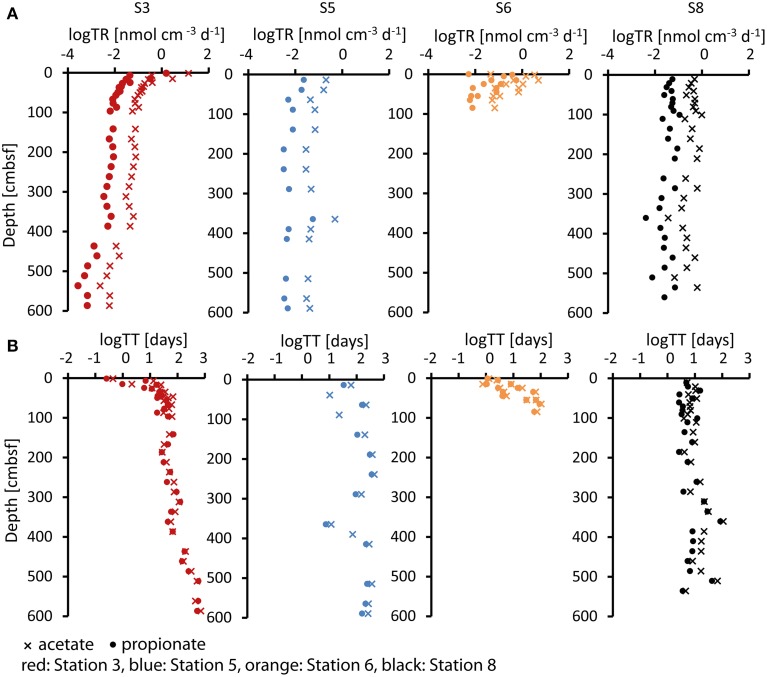
**(A)** Log turnover rates (log TR) in (nmol cm^−3^ d^−1^) and **(B)** log turnover times (log TT) in (days) of acetate (cross) and propionate (dot) at the four stations calculated from sulfate reduction rates as described in the text.

Turnover rates for acetate and propionate were calculated from sulfate reduction rates (Figure 3) as described above and are shown in Figure 5A. Acetate turnover rates were approximately nine-fold faster than those for propionate (which is the ratio between 40 and 8% divided by the stoichiometry 0.57:1). At Station 3, sulfate reduction rates and thus estimated acetate and propionate turnover rates decreased strongly downcore, starting in the uppermost sample with rates of 14 nmol cm^−3^ d^−1^ for acetate and 1.6 nmol cm^−2^ d^−1^ for propionate. With a decrease of nearly four orders of magnitude, Station 3 covered the widest range of acetate and propionate turnover rates of all four sites. Rates at Station 5 were relatively low in the uppermost samples (acetate = 0.2 nmol cm^−3^ d^−1^, propionate = 0.024 nmol cm^−3^ d^−1^) and decreased over only one order of magnitude. Station 6 showed a decrease over two orders of magnitude within the upper 100 cmbsf (acetate = 5 nmol cm^−3^ d^−1^, propionate = 0.55 nmol cm^−3^ d^−1^). Below 100 cm depth, sulfate reduction rates were below detection and VFA turnover rates could not be calculated. Station 8 showed relatively constant VFA turnover rates throughout the core, similar to Station 5 but at rates approximately twice as high (uppermost samples: acetate = 0.49 nmol cm^−3^ d^−1^, propionate = 0.056 nmol cm^−3^ d^−1^). In these calculations we have assumed a complete oxidation of propionate to bicarbonate. However, if propionate is fermented to acetate and H_2_, as discussed in Sørensen et al. (1981), a proportion of the turnover calculated for acetate would additionally account for propionate turnover.

Relative to the calculated VFA turnover rates due to sulfate reduction, which cover a broad span over four orders of magnitude, the *in-situ* porewater concentrations are much more constant throughout the cores. This suggests that the *in-situ* VFA concentrations are not directly controlled by the kinetics of their turnover.

### Diffusion and VFA turnover time

Diffusion of VFAs could, in theory, represent a bottleneck for the uptake of the substrates if the diffusion time between the cells generating the VFAs (e.g., fermenters) and the cells utilizing these VFAs as substrates (e.g., sulfate reducers) were long compared to the substrate turnover time. The measured VFA concentrations represent the average concentration in the porewater retrieved from a few cm^3^ of sediment. On a cell to cell scale, however, the VFAs diffuse from producer cells to consumer cells and this diffusion must be associated with local gradients. To evaluate the potential for diffusion limitation we compared mean diffusion times of VFAs between neighboring cells with the turnover times of the VFA pools.

The diffusion coefficient (*D*) for acetate in aqueous dilutions was calculated to be 1.95 × 10^−5^ cm^3^ s^−1^ at 2°C (Leaist and Lyons, 1984). We used this value of *D* to calculate the mean diffusion time (*t*) of acetate over a mean distance between neighboring cells (*L*) by a modified *Einstein-Smoluchowski* relation (Sten-Knudsen, 1995; Jørgensen et al., 2004) (Equation 5),

(5) $$\begin{array}{l} t=\frac{\pi L^{2}}{4D} \end{array}$$

Assuming an even distribution of cells, we calculated the mean distance between cells (*L*) (Figure 4B) from the cell abundance in the sediment (Figure 4A). At Stations 3 and 6, the average neighboring cell distance increases from 10 μm at the top to 30 μm at the bottom of the cores. The relatively constant cell numbers with depth at Station 5 and 8 result in average neighboring cell distances of 13–20 μm. Calculated diffusion times for acetate between the neighboring cells at Stations 3 and 6 range from 0.03 s at the surface to 0.4 s at the bottom of the core (Figure 4C), while at Stations 5 and 8 mean diffusion times were around 0.1 s.

The assumptions of even cell distribution and diffusion only between neighboring cells are obviously over-simplified. However, if we assume a 3–10-fold greater distance between fermenters and sulfate reducers, about 100 μm, the acetate diffusion time would still be below 4 s. With an even distribution of cells this would correspond to 10% sulfate reducers (Hoehler and Jørgensen, 2013) and 1% fermenters, which is probably an underestimate.

By dividing the *in-situ* acetate and propionate concentrations in the porewater (Figure 2) by their estimated turnover rates (Figure 5A), the turnover times of the acetate and propionate pools were calculated (Figure 5B). Turnover times at Station 3 increased with depth over a wide range from 10 h to 3–4 years for acetate and 7 h to 3 years for propionate. Turnover times at Station 5 increased from 2 months to 3 years for acetate and from 1 to 8 months for propionate. At Station 6, turnover times increased from 1 day at the surface to 5 months below 100 cmbsf below which sulfate reduction was below the detection limit. Fastest turnover times for acetate and propionate at depth were observed at Station 8.

For the calculation of turnover rates and times we used the estimated contributions of acetate and propionate of 40 and 8%, respectively, published by Finke et al. (2006). Even when taking a higher contribution of these acids, 65 and 14%, as published by Christensen (1984), turnover times would still be in a similar range, e.g., between 7 h and 2 years for acetate and between 5 h and 2 years for propionate in Station 3, thus covering the full range of turnover times represented by our estimates.

The very long turnover times of acetate and propionate, ranging from days to years, are in stark contrast to the calculated mean diffusion times of acetate between cells, which are in the range of seconds. This shows that diffusion is relatively a very fast process and that the microbial cells are surrounded by dilute substrates at constant steady-state concentrations and slow turnover. Hence, it is strongly indicated that diffusion does not limit the turnover of VFAs and that, in turn, the VFA concentrations are not the result of diffusion-limited uptake.

Similar conclusions were obtained for samples from ODP Leg 201, Site 1226. In the deep sediment cores of Site 1226 (up to 400 mbsf) acetate turnover times were estimated to be 24 years (Wang et al., 2010). Here we demonstrate that even in much shallower sediments with shorter VFA turnover times diffusion is not controlling the uptake or concentrations of VFAs.

### Gibbs energy of sulfate reduction

The energy that is available to microorganisms by a specific catabolic reaction is expressed by the Gibbs energy of the chemical reaction (ΔG_r_) which is constrained by the chemical environment in which the reaction takes place, namely the concentrations of educts and products, as well as the *in-situ* temperature and pressure. Standard Gibbs energy of the reactions, ${\Delta \text{G}}_{\text{r}}^{0}$, were calculated using standard state (i.e., activities of all compounds = 1 mol L^−1^) at the *in-situ* pressure and temperature as described above. For comparison, we also calculated the ${\Delta \text{G}}_{\text{r}}^{0}$ at the reference pressure and temperature conditions (*p* = 1 bar = 0.1 MPa, *T* = 25°C = 298 K). ${\Delta \text{G}}_{\text{r}}^{0}$ values were calculated for sulfate reduction utilizing formate (Equation 6), acetate (Equation 7), propionate (Equation 8) and, for comparison, also butyrate (Equation 9) as electron donor.

(6) $$\begin{array}{r} \text{Formate}:\;4\;HCOO^{-}+SO_{4}^{2-}+H^{+}\to HS^{-}+4\;HCO_{3}^{-} \end{array}$$

(7) $$\begin{array}{r} \text{Acetate}:\;CH_{3}COO^{-}+SO_{4}^{2-}\to {HS}^{-}+2\;HCO_{3}^{-} \end{array}$$

(8) $$\begin{array}{r} \text{Propionate}:\;4\;C_{2}H_{5}COO^{-}+7\;SO_{4}^{2-}\to 7\;HS^{-}\\ \;+\;12\;HCO_{3}^{-}+H^{+} \end{array}$$

(9) $$\begin{array}{r} \text{Butyrate}:\;2\;C_{3}H_{7}COO^{-}+5SO_{4}^{2-}\to 5\;HS^{-}+8HCO_{3}^{-}+H^{+}\; \end{array}$$

Standard Gibbs energy of sulfate reduction per mole VFA at reference temperature and pressure increase with increasing number of carbon atoms in the individual VFAs [Equation 6: −46.9 kJ (mol formate)^−1^, Equation 7: −48.1 kJ (mol acetate)^−1^, Equation 8: -74.0 kJ (mol propionate)^−1^, Equation 9: -102.5 kJ (mol butyrate)^−1^]. The difference in ${\Delta \text{G}}_{r}^{0}$ between formate and acetate is small. The recalculation to high *in-situ* pressure and low *in-situ* temperature leads to a decrease in standard Gibbs energy of reaction [Equation 6: −46.6 kJ (mol formate)^−1^, Equation 7: −44.5 kJ (mol acetate)^−1^, Equation 8: −67.7 kJ (mol propionate)^−1^, Equation 9: −93.6 kJ (mol butyrate)^−1^]. The relative effect increases with increasing number of carbon atoms in the VFAs resulting in significantly less negative ${\Delta \text{G}}_{\text{r}}^{0}$ values at *in-situ T* and *p* conditions for all acids except for formate where the change is negligible. The differences of the *T* and *p* corrected values and the values at reference *T* and *p* are mainly due to the temperature difference between 25 (reference temperature) and 2°C (*in-situ* temperature). The influence of increasing pressure over the *in-situ* range has only negligible effect on the ${\Delta \text{G}}_{\text{r}}^{0}$ values. Higher *in-situ* temperatures would thus result in higher Gibbs energy values. However, sub-arctic fjord sediments were exposed to constant low temperatures of 2°C and do not show seasonal variations. As a result the system can be considered stable over long time periods and the measured concentrations and calculated Gibbs energies reflect a long-term equilibrium.

**Figure 6:**
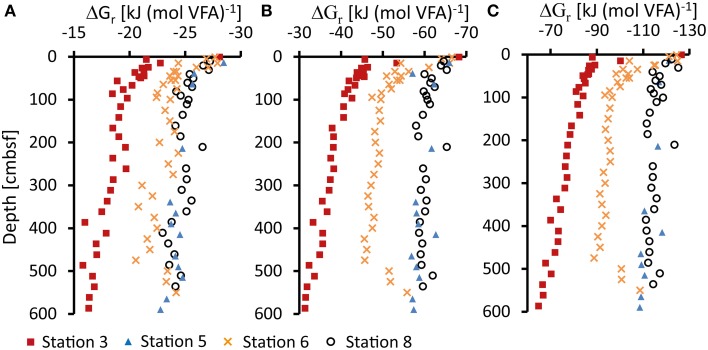
**Gibbs energy for sulfate reduction (ΔG_***r***_) in kJ (mol VFA)^−1^ at 2°C (273 K) calculated from measured VFAs (A, formate; B, acetate; C, propionate), sulfate, sulfide, and bicarbonate (DIC) concentrations and calculated activity coefficients γ**. pH was set to 7 for all calculations. See text for details.

Gibbs energy of organoclastic sulfate reduction under *in-situ* conditions (ΔG_r_) in the sediment were calculated from Equation (2) using porewater concentrations of VFAs (Figure 2), sulfate, DIC, and hydrogen sulfide (Figure 2). For the calculation of ΔG_*r*_ from formate and propionate a pH of 7 was assumed. Calculated ΔG_r_ values of *in-situ* sulfate reduction utilizing different VFAs as electron donors are shown in Figure 6. The data are expressed in kJ (mol VFA)^−1^ (ΔG_r, i_) according to Equation (4), i.e., per mol formate (Figure 6A), acetate (Figure 6B) or propionate (Figure 6C). The ΔG_r_ becomes more negative, corresponding to a larger molar energy yield, with increasing number of carbon atoms in the VFAs. At all stations, the Gibbs energy of sulfate reduction utilizing formate, actetate or propionate becomes less negative down-core, mainly as a result of increasing DIC and decreasing sulfate concentrations. Energy gained from sulfate reduction ranged from −28 to −16 kJ mol^−1^ for formate, −68 to −31 kJ mol^−1^ for acetate and −126 to −65 kJ mol^−1^ for propionate from top to bottom of the cores.

Sulfate reduction at the shelf Station 3 showed a strong decrease in free energy yield with depth, most steeply near the sediment surface and covering the whole range of ΔG_r_ values calculated for the three VFAs (Figure 3). Within the top 0–5 cm at Station 6, ΔG_r_ was similar to Station 3, −28 kJ mol^−1^ for formate, −67 kJ mol^−1^ for acetate, and −125 kJ mol^−1^ for propionate, and the free energy decreased with depth in the upper 100 cmbsf. Below this depth, free energy yields became only slightly less negative with depth as a result of the rather constant concentrations of sulfate and DIC (Figure 2). This coincides with the observations that sulfate reduction rates could not be measured experimentally below this depth. Absolute ΔG_r_ values at Stations 5 and 8 decreased only slightly with depth showing values around −24 kJ mol^−1^ for formate, −60 kJ mol^−1^ for acetate and −115 kJ mol^−1^ for propionate. In these sediments the catabolic reaction products (i.e., sulfide and DIC) have lower concentrations and consequently Gibbs energy of the catabolic reaction was higher (more negative). Propionate concentrations in the sediment were an order of magnitude lower and propionate oxidation had a significantly more negative ΔG_r_. The oxidation of butyrate was energetically even more favorable and butyrate concentrations were generally below detection limit in the sediment. However, as already discussed, if propionate and butyrate were further fermented to acetate and H_2_ their low concentrations in the porewater could be controlled by the fermenters rather than by the sulfate reducers.

A recently published approach to evaluate the available energy in subsurface environments is the combination of the ΔG_r_ values with the concentrations of the substrate present in the environment (energy density). By multiplication of ΔG_r_ with the substrate concentrations energy densities can be expressed in J (kg H_2_O)^−1^ (LaRowe and Amend, 2014; Osburn et al., 2014). This provides a metric which can relate the energetics of potential microbial processes to the environmental conditions or physiologies. Because the VFA concentrations in the porewater were generally constant with depth and also similar between the individual stations, energy density profiles follow the Gibbs energy profiles shown in Figure 6. However, due to the low concentrations of propionate compared to formate and acetate, the energy density provided by acetate was highest in the sediments [decreasing with depth from 0.4 to 0.06 J (kg H_2_O)^−1^], followed by formate [0.1–0.02 J (kg H_2_O)^−1^], and propionate [0.05–0.01 J (kg H_2_O)^−1^]. This illustrates the dominant importance of acetate as a substrate for sulfate reduction in the sediments.

### Energy limitation

It is a fundamental requirement that the catabolic reactions provide sufficient energy to enable the organisms to generate ATP which is the energy currency in living cells (Thauer et al., 1977; Schink, 1997). The synthesis of ATP in cells requires about 50 kJ mol^−1^ (Thauer et al., 1977). A fraction of the energy invested in ATP generation is lost as heat. This energy dissipation is estimated to be 20 kJ (mol ATP)^−1^, resulting in a minimum energy requirement for the synthesis of one mole of ATP of 70 kJ (Schink, 1990). Even under energy limitation (where cells are believed to reduce the heat loss to a minimum) at least 60 kJ (mol ATP)^−1^ may likely be required (Schink, 1997). ATP synthesis itself is coupled to the transport of charged ions, usually protons but also sodium, across the cell membrane (Mitchell, 1966). The general theory is that three protons cross the membrane for the generation of one molecule of ATP (Ferguson and Sorgato, 1982; Maloney, 1983). This results in a minimum free energy requirement of -20 kJ (mol proton)^−1^ to maintain a living cell (Schink, 1990, 1997). This theoretical concept was tested for hydrogen consuming microorganisms in anoxic sediments from Cape Lookout Bight, i.e., for sulfate-reducing bacteria or, in the absence of sulfate, for methane-producing Archaea (Hoehler et al., 2001). The calculated free energy yields for hydrogen consuming sulfate reduction were found to decrease with depth until they reached an asymptote of −19.1 kJ (mol sulfate)^−1^, which is in accordance with the theoretical concept. For hydrogen-driven methanogenesis an asymptote was found at −10.6 kJ (mol CO_2_)^−1^.

A similar asymptote to a certain energy yield for organoclastic sulfate reduction was not observed in our study (see Figure 4A). All calculated ΔG_*r*_ values per mol sulfate (or per mol acetate) exceeded (i.e., were more negative than) the −20 kJ mol^−1^ limit. However, hydrogen uptake in cells is different from the uptake of VFAs as hydrogen can freely diffuse though the cell membrane whereas VFA anions require an active uptake. Such an uptake system might require additional energy for the transport of VFAs which could result in a higher energy limit for organoclastic sulfate reduction. Additionally, molecular activation of VFAs (e.g., Schauder et al., 1986) might require additional energy.

To the best of our knowledge the acetate uptake mechanism in microbes from the deep biosphere is unknown and chemostat experiments with pure cultures are required to elucidate such mechanistic details. For acetoclastic sulfate reduction in *Desulfobacter* spp. Rabus et al. (2006) estimated that up to 2/3 of a mol ATP is synthesized by reducing one mole of sulfate. For an estimated 50 kJ mol^−1^ needed to generate 1 mole ATP from ADP (Thauer et al., 1977; Schink, 1997) this would result in a minimum energy requirement of −33 kJ (mol sulfate)^−1^ (or acetate) for acetoclastic sulfate reduction. This is in accordance with findings by Jin and Bethke (2009) in batch reactor experiments with *Desulfobacter hydrogenophilus* (DSM3380) that consumed acetate and sulfate. In their experiment sulfate and acetate decreased during 6 days of incubation and the available energy leveled off at −33.1 to −42.8 kJ mol^−1^. It is striking that this level is similar to what we observe at the bottom of Station 3 (−31 kJ mol^−1^). At this depth, sulfate is depleted to 240 μM (Figure 2) and acetate turnover rates have dropped to 0.01 nmol cm^−3^ d^−1^ (Figure 5A). Thus, sulfate reduction might indeed proceed at the energetic limit in the sediment. In contrast, at Station 6, sulfate reduction is already below detection limit at 100 cmbsf (Figure 3). The Gibbs energy of sulfate reduction from acetate is, however, with −49 kJ mol^−1^ still relatively high, considerably higher than what we observe at the bottom of Station 3 (Figure 6). Furthermore, it is obvious that the low turnover rates observed at Station 5 are not a result of low energy yield of the reactions as ΔG_r_ is significantly above the lowest ΔG_r_ observed at the bottom of Station 3.

## Conclusion

The aim of the current study was to understand the *in-situ* controls in sulfate reducing sediments on the concentrations and turnover of porewater VFAs, which represent key intermediates in the anaerobic microbial food chain. For this purpose, we analyzed porewater concentrations of sulfate, sulfide, DIC, and VFAs, as well as cell abundance and sulfate reduction rates. VFA concentrations were low and surprisingly constant with depth and sediment age, suggesting that the concentrations reflect a tightly controlled steady state. In sediments with very low rates of sulfate reduction, below our experimental detection limit, concentrations seemed to be less well constrained. We suggest that these VFA data represent threshold concentrations below which the microorganisms are unable to take up and metabolize the VFAs.

The observation that the steady-state concentrations of VFAs were similar over a very broad range of turnover rates suggests that they were not kinetically controlled. Calculated turnover times of acetate and propionate by sulfate reduction vastly exceeded diffusion times between the cells in the sediments. Consequently, cells lived in a uniform and constant, dilute solution of VFAs. Diffusion was not limiting the uptake of VFAs and VFA concentrations were not controlled by VFA diffusion. Gibbs energy of VFA dependent sulfate reduction in the sediments decreased with depth throughout the sulfate zone. Propionate was the most energy yielding substrate per mol among the three VFAs. Propionate concentrations were an order of magnitude lower than formate and acetate concentrations. A potential energetic limit of acetoclastic sulfate reduction might be reached at a ΔG_r_ of approximately −30 kJ (mol acetate)^−1^ at the bottom of the shelf Station 3.

It still remains unclear what exactly controls the VFA concentrations in the sediment porewater. It is likely that physiological constraints, perhaps associated with VFA uptake and activation, determine the *in-situ* porewater concentrations and that utilization below certain threshold concentrations is not feasible. These concentrations, together with the concentrations of the other substrates and products, determine the minimum ΔG_r_ for organoclastic sulfate reduction in the sediment. Consequently, this minimum energy would be different for different microbial communities (e.g., in sulfate reducing and methanogenic communities) and also different for individual substrates.

## Author contributions

CG, HR, and BJ designed the study; MJ, HR, and BL collected the samples; BL curated the cores and led the sampling; MS led the cruise; CG and MJ performed the laboratory work; CG and MJ wrote the paper; HR and BJ edited the paper; all authors reviewed the manuscript.

### Conflict of interest statement

The authors declare that the research was conducted in the absence of any commercial or financial relationships that could be construed as a potential conflict of interest.
