# Supplementary material for: Formate, acetate, and propionate as substrates for sulfate reduction in sub-arctic sediments of Southwest Greenland
Source: Front Microbiol. 2015 Aug 24;6:846. doi: 10.3389/fmicb.2015.00846 (PMC4547046; doi:10.3389/fmicb.2015.00846)
Supplement: Supplementary file 1 [file Table1.DOCX]

***Supplementary Material***

**Formate, acetate and propionate as substrates for sulfate reduction in sub-arctic sediments of Southwest Greenland.**

**Clemens Glombitza*, Marion Jaussi, Hans Røy, Marit-Solveig Seidenkrantz, Bente Aagaard Lomstein, Bo Barker Jørgensen**

*****Corresponding Author: clemens.glombitza@bios.au.dk

1. **Supplementary Data**
2. **Supplementary Figures and Tables**

## Supplementary Figures

## Supplementary Tables

## Supplementary Table 1. Thermodynamic properties used for the calculation of standard state Gibbs energy change of reaction using SUPCRT92/OBIGT software. References: a) Schock, 1995, b) Schock and Helgeson, 1988, and c) Schock et al. 1997.

| **Species** | **γ** | **G_f_^0^** | **H_f_^0^** | **S** | **V** | **C_p_** | **Reference** |
| --- | --- | --- | --- | --- | --- | --- | --- |
|  | T=275 K I=0.7 | [kJ mol^-1^] | [kJ mol^-1^] | [J mol^-1^ K^-1^] | [cm^3^ mol^-1^] | [J mol^-1^ K^-1^] |  |
| CHO_2_^-^ | 0.67 | -350.88 | -425.43 | 90.79 | 25.76 | -83.85 | a) |
| C_2_H_3_O_2_^-^ |  | -369.32 | 486.10 | 86.19 | 40.10 | 26.99 | a) |
| C_3_H_5_O_2_^-^ |  | -363.05 | -513.08 | 110.88 | 54.58 | 136.31 | a) |
| C_4_H_7_O_2_^-^ |  | -354.18 | -538.19 | 133.05 | 69.95 | 186.69 | a) |
| SO_4_^2-^ | 0.17 | -744.46 | -909.60 | 18.83 | 12.92 | -266.10 | b) |
| HS^-^ | 0.63 | 11.97 | -16.11 | 68.20 | 20.35 | -93.93 | b) |
| HCO_3_^-^ | 0.67 | -586.94 | -689.93 | 98.45 | 24.21 | 34.85 | b) |
| H^+^ | - | 0 | 0 | 0 | 0 | 0 | c) |
